# High burden of geriatric assessment impairments across the adult age spectrum in patients with cancer

**DOI:** 10.1093/oncolo/oyag010

**Published:** 2026-01-12

**Authors:** Chin-Tung Nien, Chieh-Ying Chang, Chang-Hsien Lu, Kun-Yun Yeh, Yu-Shin Hung, Wen-Chi Chou

**Affiliations:** Department of Hematology and Oncology, Chang Gung Memorial Hospital at Linkou and College of Medicine, Chang Gung University, Taoyuan 333, Taiwan; Department of Hematology and Oncology, Chang Gung Memorial Hospital at Linkou and College of Medicine, Chang Gung University, Taoyuan 333, Taiwan; Department of Hematology and Oncology, Chang Gung Memorial Hospital at Chiayi, Chiayi 600, Taiwan; Department of Hematology and Oncology, Chang Gung Memorial Hospital at Keelung, Keelung 204, Taiwan; Department of Hematology and Oncology, Chang Gung Memorial Hospital at Linkou and College of Medicine, Chang Gung University, Taoyuan 333, Taiwan; Department of Hematology and Oncology, Chang Gung Memorial Hospital at Linkou and College of Medicine, Chang Gung University, Taoyuan 333, Taiwan

**Keywords:** age factors, geriatric assessment, neoplasm, prevalence, polypharmacy

## Abstract

**Background:**

The age-specific distribution of frailty and its domain-level characteristics remain poorly understood across the adult population of patients with cancer. We aimed to quantify the frailty prevalence and geriatric assessment (GA) impairment patterns in adults and to examine their prognostic relevance in newly diagnosed patients with cancer.

**Material and Methods:**

This multicenter, cross-sectional cohort enrolled 2,501 adults (≥20 years) before therapy in 2021-2023. GA covered 8 domains (function, comorbidity, cognition, mood, nutrition, polypharmacy, falls, and social support). Patients were grouped into 6 age bands (20-39 to ≥80) and labeled fit (0 deficits), prefrail (1), or frail (≥2). We analyzed age-specific geriatric impairment patterns and overall survival (OS).

**Results:**

The mean number of GA deficits increased significantly across the 6 ordered age bands. Frailty was common and increased with age: 40.0% (20-39), 42.3% (40-49), 56.2% (70-79), and 74.4% (≥80). Malnutrition was the most frequent deficit (59.1% overall), affecting 51% of patients aged 20-39 years, peaking at 63.8% in the 70-79-year cohort. Older age groups showed steeper increases in comorbidities, cognitive impairment, and functional decline. Polypharmacy and depressed mood were frequent, but varied less with age; inadequate social support was uniformly low. In multivariable models, prefrailty and frailty predicted worse OS. Age-stratified analyses of 40-49, 50-59, and 70-79-year cohorts showed similar associations.

**Conclusion:**

Frailty is prevalent across adults of all ages with distinct, age-associated GA profiles. Nutritional deficits were the most prevalent impairment even among younger adults, whereas functional, comorbidity, and cognitive burdens escalate in older patients with cancer. Routine pretreatment GA for all adults can identify vulnerabilities and enable age-tailored supportive interventions.

## Graphical abstract

**Graphical Abstract:**
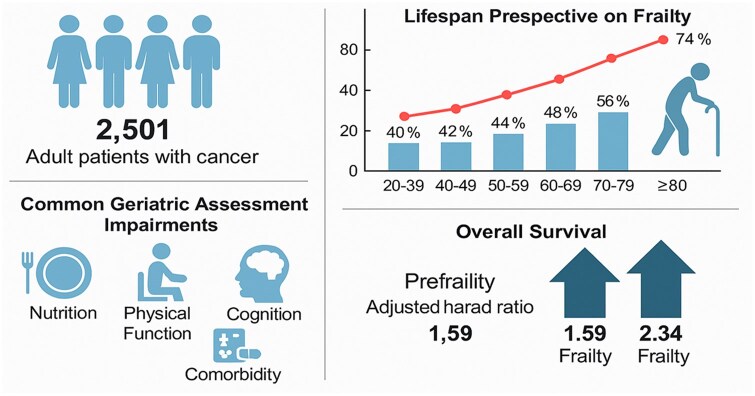

Implication for Practice This large multicenter study demonstrates that frailty and geriatric assessment (GA) impairments are highly prevalent not only in older adults but also in younger and middle-aged patients with newly diagnosed cancer. Distinct age-specific vulnerability patterns, nutritional deficits in younger adults and functional, cognitive, and comorbidity burdens in older adults, highlight the limitations of relying solely on chronological age to guide supportive care. Because frailty and prefrailty independently predict poorer survival across most age groups, integrating routine pretreatment GA for all adults can facilitate earlier identification of vulnerabilities and enable age-tailored interventions to improve treatment tolerance and outcomes.

## Introduction

Frailty is a complex clinical syndrome characterized by reduced physiological reserves and increased vulnerability to stressors.^1^ In the context of cancer, this vulnerability translates into significant decline in physical, cognitive, and/or social functioning and ultimately diminishes the quality of life of cancer survivors.^2^ Extensive research has consistently revealed a strong association between frailty and adverse outcomes in patients with cancer, including increased disabilities, morbidity rates, surgical complications, and elevated mortality.^3–6^ Therefore, frailty represents a critical multidimensional syndrome with profound prognostic implications for individuals undergoing cancer treatment.

Traditionally, frailty has been predominantly associated with older age; however, emerging data have revealed that younger adults with cancer exhibit phenotypes similar to accelerated biological aging.^7^ Clinical evidence from survivors of hematologic malignancies and solid tumors further supports the presence of functional and cognitive impairments, sarcopenia, and poor health-related quality of life in non-older cancer cohorts.^8^^,^^9^ These findings suggest that the current age-based cut-off for frailty screening may overlook a significant subset of vulnerable patients.

Geriatric assessment (GA) is a multidimensional evaluation of medical, functional, physical, psychological, and socioenvironmental domains used to identify vulnerabilities and guide treatment and supportive care in oncology.^10^^,^^11^ Frailty is commonly defined by either the Fried physical phenotype or the deficit-accumulation model, which reflects the burden of impairments across multiple domains.^2^ GA-based approaches align more closely with the deficit-accumulation framework, capturing multidimensional frailty and identifying overlapping but distinct frail populations compared with phenotype-based definitions. While the ASCO recommends routine use for patients aged ≥65 years, there is a notable lack of empirical justification for this age cutoff. In fact, the relationship between chronological age and GA-detected impairment remains inadequately defined in patients under 65 years of age.^12^^,^^13^ Consequently, opportunities for the early detection of frailty and intervention may be missed in younger patients who are vulnerable but asymptomatic or misclassified as “fit” based on age alone.

To date, most studies concerning frailty in oncology have focused on older adults or broad age groups, which limits the resolution of age-specific trends. Prior studies, such as those by Pergolotti et al. and Giri et al., provided important early insights into the prevalence of frailty in populations younger than 64 years but were constrained by small sample sizes, single-institution designs, and restricted cancer types or age ranges.^12^^,^^13^ Therefore, the age-specific distribution of frailty and its GA domain characteristics remain poorly understood in the wider adult cancer population.

Addressing this knowledge gap is essential to refine frailty screening guidelines, individualize treatment strategies, and improve patient outcomes. To overcome these limitations, we conducted a multicenter, cross-sectional study that involved 2,501 adult patients with newly diagnosed cancer stratified into 6 age groups (20-80 years). Our objectives were to (1) estimate the prevalence of frailty across the adult age continuum using standardized GA tools, (2) characterize the age-specific distribution of GA domains, and (3) evaluate the association between frailty status and overall survival. By providing granular, age-stratified data on the frailty burden and prognostic impact, this study provided a more inclusive, functionally driven framework for frailty screening in cancer care.

## Materials and methods

### Study design and population

This cross-sectional observational study was conducted at 3 medical centers in Taiwan between January 2021 and December 2023. Eligible patients met the following criteria: (1) age ≥20 years; (2) pathologically confirmed diagnosis of cancer; (3) scheduled to receive antitumor treatment (e.g., surgery, chemotherapy, radiotherapy, or combined modalities); and (4) completed baseline GA within 7 days before treatment initiation. Patients who had previously received cancer treatment were excluded, and all the study participants provided written informed consent. The study protocol was approved by the Institutional Review Board of Chang Gung Memorial Hospital (IRB No. 202501211B0), and all procedures were conducted in accordance with the Declaration of Helsinki.

### Geriatric assessment

Frailty status was evaluated using a standardized battery that covered 8 domains: functional status (activities of daily living [ADL] and instrumental ADL), comorbidity burden (modified Charlson Comorbidity Index, with exclusion of age and cancer diagnosis), fall history (self-reported falls in the past 6 months), cognitive function (Mini-Mental State Examination), mood (Geriatric Depression Scale-4), nutritional impairment was defined as a Mini Nutritional Assessment-Short Form (MNA-SF) score ≤11, encompassing both patients at risk of malnutrition (scores 8-11) and those with overt malnutrition (scores ≤7), polypharmacy (concurrent use of ≥5 medications daily), and social support. All comorbidities were collected at baseline by trained research personnel using standardized data abstraction procedures to ensure consistency across participating centers. Each domain was scored and dichotomized by established cutoffs as previously described,^14^ and the total number of impaired domains (range 0-8) was used to define frailty status: fit (0 deficits), prefrail (1 deficits), or frail (≥2 deficits). GA was conducted at baseline within 7 days prior to initiation of antitumor treatment. All assessments were performed in person during outpatient clinic visits. The GA was administered by trained research nurses or study coordinators using standardized assessment protocols. Patient-reported components were completed through structured interviews, while performance- and record-based domains were verified through direct assessment and medical record review.

### Outcome measures

The primary outcome was frailty prevalence across the predefined age groups (20-39, 40-49, 50-59, 60-69, 70-79, and ≥80 years). The secondary outcomes included (1) the distribution of individual domain impairments and the correlation between chronological age and frailty status and (2) the association between frailty status and overall survival within each age stratum.

### Statistical analysis

Statistical analyses were performed to examine associations and compare outcomes across the defined age groups. For baseline characteristics, descriptive statistics were presented across 6 age strata, while formal statistical comparisons were performed only for dichotomized age groups (<65 vs ≥65 years) to enhance interpretability. Hazard ratios (HR) were generated to assess the differences in overall survival among frailty statuses, with comparisons made using Cox regression analysis. The adjusted HR in the multivariate Cox regression models were calculated after adjusting for sex, educational level, marital status, cancer type, tumor stage, and treatment modality. Statistical significance was set at a 2-sided *P*-value of <.05.

## Results

### Patient characteristics

The demographic and clinical characteristics of the diverse cohort of 2,501 patients with cancer are detailed in Table 1. The study population was stratified into 6 distinct age groups to allow for a granular analysis of age-related trends: 100 patients (4.0%) aged 20-39 years, 260 patients (10.4%) aged 40-49 years, 510 patients (20.4%) aged 50-59 years, 795 patients (31.8%) aged 60-69 years, 660 patients (26.4%) aged 70-79 years, and 176 patients (7.0%) aged ≥80 years.

**Table 1.** Distribution of baseline characteristics across different age groups, *n* (%).

Variable	All	Age group (years)					
		20-39	40-49	50-59	60-69	70-79	≥80
**Patient number**	2501 (100)	100 (4.0)	260 (10.4)	510 (20.4)	795 (31.8)	660 (26.4)	176 (70.)
**Sex**							
** Male**	1664 (66.5)	70 (70.0)	190 (73.1)	389 (76.3)	515 (64.8)	392 (59.4)	108 (61.4)
** Female**	837 (33.5)	30 (30.0)	70 (26.9)	121 (23.7)	280 (35.2)	268 (40.6)	68 (38.6)
**Marriage**							
** Married**	2013 (80.5)	64 (64.0)	185 (71.2)	409 (80.2)	692 (87.0)	538 (81.5)	125 (71.0)
**Educational level**							
** Nil or elementary school**	779 (31.1)	3 (3.0)	10 (3.8)	39 (7.6)	258 (32.5)	359 (54.4)	110 (62.5)
** High school**	1228 (49.1)	46 (46.0)	162 (62.3)	359 (70.4)	416 (52.3)	201 (30.5)	44 (25.0)
** College**	451 (18.0)	48 (48.0)	75 (28.8)	103 (20.2)	112 (14.1)	93 (14.1)	20 (11.4)
** Higher than college**	43 (1.7)	3 (3.0)	13 (5.0)	9 (1.8)	9 (1.1)	7 (1.1)	2 (1.1)
**Main caregiver**							
** Partner/spouse**	1187 (47.5)	32 (32.0)	144 (55.4)	295 (57.8)	429 (54.0)	246 (37.3)	41 (23.3)
**Current working status**							
** Working**	1107 (44.3)	84 (84.0)	214 (82.3)	377 (73.9)	309 (38.9)	107 (16.2)	16 (9.1)
**Current or previous drinking**							
** Yes**	832 (33.3)	33 (33.0)	122 (46.9)	249 (48.8)	238 (29.9)	158 (23.9)	32 (18.2)
**Current or previous smoking**							
** Yes**	985 (39.4)	41 (41.0)	144 (55.4)	290 (56.9)	296 (37.2)	173 (26.2)	41 (23.3)
**Cancer type**							
** Head and neck**	573 (22.9)	44 (44.0)	127 (48.8)	208 (40.8)	146 (18.4)	42 (6.4)	6 (3.4)
** Esophagus**	217 (8.7)	4 (4.0)	39 (15.0)	87 (17.1)	62 (7.8)	22 (3.3)	3 (1.7)
** Thorax**	118 (4.7)	3 (3.0)	9 (3.5)	32 (6.3)	40 (5.0)	31 (4.7)	3 (1.7)
** Breast**	113 (4.5)	2 (2.0)	10 (3.8)	11 (2.2)	51 (6.4)	37 (5.6)	2 (1.1)
** Stomach or small bowel**	381 (15.2)	8 (8.0)	22 (8.5)	42 (8.2)	123 (15.5)	135 (20.5)	51 (29.0)
** Pancreas**	213 (8.5)	3 (3.0)	7 (2.7)	29 (5.7)	80 (10.1)	84 (12.7)	10 (5.7)
** Liver**	247 (9.9)	0	13 (5.0)	28 (5.5)	85 (10.7)	93 (14.1)	28 (15.9)
** Colorectal**	316 (12.6)	2 (2.0)	4 (1.5)	24 (4.7)	110 (13.8)	131 (19.8)	45 (25.6)
** Hematologic**	205 (8.2)	25 (25.0)	20 (7.7)	33 (6.5)	64 (8.1)	44 (6.7)	19 (10.8)
** Genitourinary**	76 (3.0)	6 (6.0)	5 (1.9)	9 (1.8)	15 (1.9)	33 (5.0)	8 (4.5)
** Others**	42 (1.7)	3 (3.0)	4 (1.5)	7 (1.4)	19 (2.4)	8 (1.2)	1 (0.6)
**ECOG status**							
** 0**	1128 (45.1)	44 (44.0)	99 (38.1)	201 (39.4)	380 (47.8)	340 (51.5)	64 (36.4)
** 1**	1175 (47.0)	50 (50.0)	150 (57.7)	276 (54.1)	351 (44.2)	265 (40.2)	83 (47.2)
** 2**	157 (6.3)	5 (5.0)	7 (2.7)	26 (5.1)	53 (6.7)	43 (6.5)	23 (13.1)
** 3**	36 (1.4)	1 (1.0)	3 (1.2)	6 (1.2)	9 (1.1)	12 (1.8)	5 (2.8)
** 4**	5 (0.2)	0	1 (0.4)	1 (0.2)	2 (0.3)	0	1 (0.6)
**Tumor stage**							
** 1**	203 (8.1)	3 (3.0)	9 (3.5)	17 (3.3)	80 (10.1)	66 (10.0)	28 (15.9)
** 2**	363 (14.5)	13 (13.0)	29 (11.2)	38 (7.5)	124 (15.6)	121 (18.3)	38 (21.6)
** 3**	580 (23.2)	9 (9.0)	59 (22.7)	108 (21.2)	163 (20.5)	191 (28.9)	50 (28.4)
** 4**	1355 (54.2)	75 (75.0)	163 (62.7)	347 (68.0)	428 (53.8)	282 (42.7)	60 (34.1)
**Treatment modality**							
** Surgery**	464 (18.6)	1 (1.0)	5 (1.9)	9 (1.8)	147 (18.5)	204 (30.9)	98 (55.7)
** Chemotherapy**	1460 (58.4)	55 (55.0)	141 (54.2)	267 (52.4)	512 (64.4)	413 (62.6)	72 (40.9)
** Concurrent chemoradiotherapy**	577 (23.1)	44 (44.0)	114 (43.8)	234 (45.9)	136 (17.1)	43 (6.5)	6 (3.4)

ECOG, Eastern Cooperative Oncology Group.

Table presents descriptive statistics only. *P*-values were omitted due to the limited interpretability of global statistical testing across multiple age strata.

Clinical characteristics varied considerably across age cohorts. The distribution of cancer types was highly significant, with head and neck cancers being markedly more prevalent in younger groups (44.0% in 20-39, 48.8% in 40-49), while stomach/small bowel, liver, and colorectal cancers prevalence varied significantly by age group, with higher proportions in older age bands (eg, colorectal: 2.0% in 20-39 vs 25.6% in ≥80). Hematologic cancers presented a bimodal distribution, peaking in the youngest (25.0% in 20–39) and oldest (10.8% in ≥80) cohorts. ECOG performance status also differed significantly; while ECOG 0 and 1 were predominant overall, higher ECOG scores (2, 3, 4) were notably more prevalent in the oldest (≥80 years) group (eg, 13.1% ECOG 2 in ≥80 vs 5.0% in 20-39). Cancer stage showed significant variation, with younger patients exhibiting a higher proportion of stage 4 disease (75.0% in 20-39, 62.7% in 40-49, 68.0% in 50-59 year cohorts), while older patients tended to have a higher proportion of stage 1 and 2 disease (eg, stage 1: 3.0% in 20-39 vs 15.9% in ≥80 year cohorts). Treatment modality also varied significantly between age groups, with concurrent chemoradiotherapy being considerably more common in younger groups (44.0% in 20-39, 43.8% in 40-49 year cohorts), whereas surgery was more prevalent in patients aged ≥80 years (55.7%).

A focused comparison between patients aged <65 and ≥65 years is provided in Table S1 (see online supplementary material), highlighting clinically meaningful age-related differences.

### Proportion of patients with geriatric domain impairment across age strata

Table 2 shows the progressive accumulation of GA deficits across 6 predefined age strata. In the youngest cohort (20-39 years), more than one quarter of patients had no impairments (26.0%) and roughly one third had a single impairment (34.0%), whereas only a small minority exhibited 4 or more deficits (≤5.0%). A similar but slightly less favorable pattern was seen in patients aged 40-49 years, among whom 20.0% were impairment-free, 37.7% had only one deficit, and fewer than 5% harbored ≥4 deficits. By contrast, the curves for the 2 oldest groups shifted markedly to the right: only 12.9% of those aged 70-79 years and 6.8% of those aged ≥80 years were free of impairments, while the ≥80-year cohort showed a pronounced “stacking” of multiple deficits, with 31.8% reporting exactly 2, 19.9% 3, 11.4% 4, 8.5% 5, and 2.8% 6 impaired domains. There was a significant correlation between the different age groups and the number of geriatric impairments (Spearman’s ρ = 0.943, *P* = .005).

**Table 2.** Proportion of patients with geriatric domain impairments across age strata.

Age group	Number of geriatric domain impairments, n (%)							
	0	1	2	3	4	5	6	Median (range)
**All patients (*n* = 2501)**	429 (17.2%)	822 (32.9%)	646 (25.8%)	362 (14.5%)	160 (6.4%)	64 (2.6%)	18 (0.7%)	1 (0–6)
**20-39 (*n* = 100)**	26 (26.0%)	34 (34.0%)	24 (24.0%)	11 (11.0%)	5 (5.0%)	0	0	1 (0–4)
**40-49 (*n* = 260)**	52 (20.0%)	98 (37.7%)	72 (27.7%)	27 (10.4%)	7 (2.7%)	4 (1.5%)	0	1 (0–5)
**50-59 (*n* = 510)**	100 (19.6%)	195 (38.2%)	123 (24.1%)	66 (12.9%)	19 (3.7%)	7 (1.4%)	0	2 (0–5)
**60-69 (*n* = 795)**	154 (19.4%)	258 (32.5%)	211 (26.5%)	102 (12.8%)	49 (6.2%)	14 (1.8%)	7 (0.9%)	2 (0–6)
**70-79 (*n* = 660)**	85 (12.9%)	204 (30.9%)	160 (24.2%)	121 (18.3%)	60 (9.1%)	24 (3.6%)	6 (0.9%)	2 (0–6)
**≥80 (*n* = 176)**	12 (6.8%)	33 (18.8%)	56 (31.8%)	35 (19.9%)	20 (11.4%)	15 (8.5%)	5 (2.8%)	2 (0–6)

### Age-related shift in frailty status

**Figure 1.**
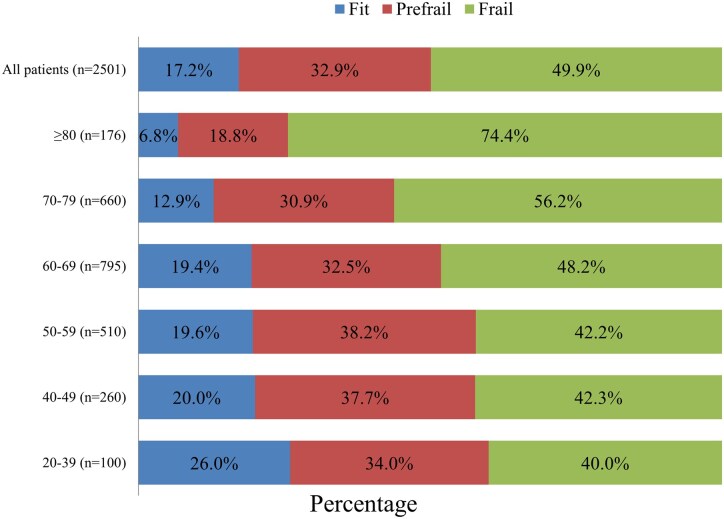
Age-stratified distribution of geriatric assessment-defined frailty. Stacked bars show the proportion of patients classified as fit (0 GA impairments), prefrail (1), or frail (≥2) overall and by age group. Frailty was prevalent across all adult age groups and increased with advancing age, from 40% in the youngest group to 74.4% in patients aged ≥80 years.

Overall, 17.2%, 32.9%, and 49.9% of patients were categorized as fit, prefrail, and frail based on GA, respectively (Figure 1). In the youngest group (20-39 years), only 40% of patients met the frailty criteria, whereas 26% were classified as fit and 34% as prefrail. The proportion of frail individuals increased steadily with each successive decade, and the incidences reached 42.3% in patients aged 40-49 years and 48.2% in those aged 60-69 years. This upward trajectory became more pronounced in the oldest cohorts: frailty affected 56.2% of patients aged 70-79 years and peaked at 74.4% among those aged ≥80 years. Conversely, the prevalence of fitness decreased from 26.0% in the youngest group to just 6.8% in the oldest. In contrast, prefrailty declined with advancing age, as a greater proportion of older patients met criteria for frailty rather than prefrailty. The overall trend underscores a significant graded association between age and cumulative vulnerability, which was corroborated by the highly significant between-group differences (*P* < .001).

In sensitivity analyses excluding the nutritional impairment domain, frailty prevalence was reduced across all age groups but remained common, including in younger adults. Frailty rates were 20.0%, 19.6%, and 25.3% in patients aged 20-39, 40-49, and 50-59 years, respectively, compared with approximately 40% in the original analysis (Table S2—see online supplementary material). A progressive age-related increase in frailty prevalence persisted, reaching 57.4% in patients aged ≥80 years.

### Prevalence of abnormal geriatric-assessment domains by age group

**Figure 2.**
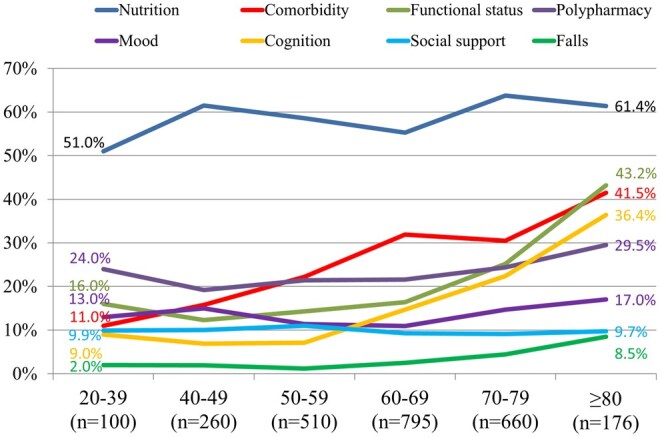
Age-specific prevalence of geriatric-assessment domain impairments. The lines depict the percentage of patients within each age stratum with impairment in the indicated GA domains. Nutritional deficits were the most prevalent problem across all age groups and increased modestly with age. However, comorbidity burden, functional limitations, cognitive deficits, and fall history increased steeply as age advanced.

Overall, the most common prevalent geriatric domain impairment was malnutrition (59.1%), followed by comorbidities (27.7%), polypharmacy (22.7%), functional decline (19.7%), cognitive impairment (15.7%), mood (13.0%), social support (9.7%), and falls (3.1%). Nutritional impairments were the most common deficit overall (59.1%) and affected one-half of patients aged 20-39 years (51.0%) and raised modestly to peak values in the 70-79-year group (63.8%; *P* = .013) **(**Figure 2**)**. In contrast, the comorbidity burden, functional limitations, and cognitive deficits showed steeper age-related escalations, while fall history also increased with age. The prevalence of a modified Charlson Comorbidity Index ≥ 2 quadrupled from 11.0% in the youngest cohort to 41.5% among those aged ≥80 years (*P* < .001), while abnormal ADL/IADL performance climbed from 16.0% to 43.2% (*P* < .001). Cognitive impairment increased nearly 4-fold, from 9.0 % in patients aged 20-39 years to 36.4% in those aged ≥80 years (*P* < .001), and falls also showed an age-associated increase (2.0% vs 8.5%, *P* < .001). Polypharmacy and depressed mood were relatively frequent, but varied less dramatically with age (22.7% and 13.0% overall, *P* = .122 and .096, respectively), whereas inadequate social support remained uniformly low (approximately 10%) across all groups (*P* = .918).

### Hazard ratios for overall survival according to frailty status within each age strata

During a median follow-up of 32.0 months (range: 0.3-46), 700 of 2,501 patients (28.0%) died, and median OS was not reached. The 1-, 2-, and 3-year OS rates were 74.6%, 61.1%, and 56.8%, respectively. In the full cohort (*n* = 2,501), compared to fit patients, both prefrail and frail groups were independently associated with a significantly increased risk of mortality, with adjusted HR values of 1.59 (95% CI: 1.05-2.34, *P* = .002) for prefrail and 2.34 (95% CI: 1.55-3.52, *P* < .001) for frail individuals. Figure S1 (see online supplementary material for a color version of this figure) presents OS by frailty status stratified by cancer type, demonstrating a consistent association between frailty and OS across diverse oncologic settings.

**Figure 3.**
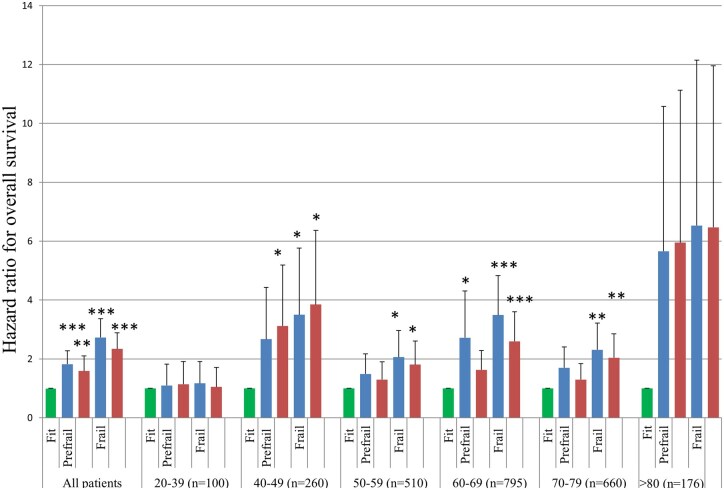
Association between frailty status and overall survival. In the overall cohort, both prefrailty and frailty were associated with a higher hazard of death compared with fit status, with unadjusted hazard ratios (blue symbols) of 1.82 (95% CI, 1.36–2.42; *P* < .001) and 2.73 (95% CI, 2.09-3.57; *P* < .001), respectively. After multivariable adjustment (red symbols), the corresponding hazard ratios were 1.59 (95% CI, 1.19-2.12; *P* = .002) and 2.34 (95% CI, 1.79-3.06; *P* < .001). In age-stratified analyses, prefrailty and frailty were generally associated with worse survival, although statistical significance was observed only in selected strata, likely reflecting limited statistical power in smaller subgroups. Error bars indicate 95% CIs. Asterisks denote levels of statistical significance (*P < .05, **P < .01, ***P < .001).

Figure 3 shows a forest plot that summarizes the univariate and multivariate Cox analyses of overall survival, with fit patients as the reference category. Stratified analyses revealed that this adverse prognostic effect of frailty was most pronounced in the 40-49, 50-59, 60-69, and 70-79-year age groups, where adjusted HRs for frail patients ranged from 1.81 to 3.85 (all *P* values <.05). Notably, in the youngest (20-39 years) and oldest (≥80 years) age groups, the association between frailty and survival was not statistically significant, which potentially reflects a limited sample size or competing risks.

In Cox regression analyses, exclusion of malnutrition attenuated the association between frailty and overall survival, particularly in younger age groups; however, frailty remained significantly associated with inferior survival in the overall cohort (HR 1.79, *P* < .001) and in older adults, with consistent directionality across age strata (Table S3—see online supplementary material).

## Discussion

This large-scale, multicenter study provides one of the most comprehensive evaluations of frailty prevalence and GA-identified impairments across the adult age continuum in patients with newly diagnosed cancer. Through enrollment of 2,501 patients aged ≥20 years and applying a standardized GA framework, we found that frailty is not confined to older patients but is also prevalent in younger adults. The prevalence of frailty and the accumulation of nutritional, functional, cognitive, and comorbidity deficits increased steadily with age. Notably, more than 40% of patients aged 20-59 years were already frail, and nearly three-quarters of those aged ≥80 years had impairments in ≥3 domain. These findings indicate that vulnerability extends beyond older adults and support frailty screening across the adult age spectrum, rather than relying solely on a fixed age-based cutoff.

The findings of the current study not only align with but substantially build upon previous smaller-cohort research findings that challenged the traditional notion of frailty as an exclusively geriatric syndrome. Prior work, such as the studies by Pergolotti et al. (*n* = 96)^13^ and Giri et al. (*n* = 455),^12^ indicated that the patterns of impairment differed between older and younger cancer adults, but these studies were limited by small sample size, broad age groupings (<65 vs ≥ 65 years), or a focus on specific cancer types.

The current study significantly expands on these earlier findings by including a much larger and more representative ­population (*n* = 2,501) across a granular age spectrum (20 to ≥ 80 years). This resolution confirms one of our most significant and novel findings: the substantial prevalence of 2 or more pretreatment GA deficits (approximately 40%), even in the youngest cancer cohort (20-39 years). This observation shifts the concept of vulnerability beyond age and suggests that the GA tool effectively captures the state of cancer-related impairment^15^ or premorbid vulnerability,^16^ which may be independent of the aging process. For patients aged 20-39 years, these deficits may reflect cancer-related metabolic and inflammatory stress rather than long-term aging processes.^17–19^ This highlights that routine pretreatment GA should not be restricted to patients over a certain age threshold (eg, 65 or 70 years). Instead, it should be adopted across the adult lifespan to identify younger patients who are equally vulnerable and require targeted supportive care interventions to mitigate treatment toxicity and improve quality of life.

A key finding of this study was that frailty was more prevalent than fitness among adults aged 20-59 years, a population not traditionally considered at high risk. In this younger cohort, frailty was driven primarily by nutritional impairment identified by the MNA-SF, rather than age-related comorbidities, functional dependence, or cognitive decline. Abnormal MNA-SF scores in younger adults likely reflect cancer-related catabolic stress, inflammation, and tumor-associated symptoms rather than chronic physiological aging.^20^^,^^21^ These results indicate that frailty in patients aged 20-59 represents a potentially reversible, disease-driven vulnerability, highlighting early nutritional assessment as an actionable target for intervention to improve treatment tolerance and outcomes.^22^^,^^23^

Further characterization of patients aged <59 years revealed substantial heterogeneity in cancer type and stage, with head and neck and esophageal cancers more prevalent and over 60% presenting with stage IV disease. Frailty was observed across diverse malignancies and stages and was not confined to indolent or early-stage disease, and its association with overall survival remained significant after adjustment for cancer type, stage, and treatment, indicating that GA-defined frailty captures clinically meaningful vulnerability beyond conventional oncologic prognostic factors. To address the potential influence of cancer site-specific malnutrition, we performed sensitivity analyses excluding the nutritional domain. Although frailty prevalence and effect sizes were attenuated, particularly in younger patients, frailty remained prevalent across age groups and continued to predict inferior survival in the overall cohort and older adults. These findings suggest that GA-defined frailty reflects multidimensional vulnerability beyond isolated nutritional impairment, while highlighting nutrition as a key and potentially modifiable contributor to cancer-related vulnerability in younger adults who may otherwise appear fit by age or performance status.

The nutritional domain of GA is involved in 2 distinct pathophysiological processes. In older adults, this impairment reflects a chronic, age-accumulated vulnerability and a lack of physiological reserves, which is consistent with the traditional frailty phenotype.^24–26^ Conversely, in the younger cohort, the high prevalence was primarily a manifestation of disease-related cachexia and catabolism, which were driven acutely by tumor-derived inflammation, regardless of the underlying age-related reserve.^27^^,^^28^ These findings suggest that frailty may reflect contributions from both aging-related vulnerability and cancer-related processes. Consequently, clinical interventions must be tailored accordingly, with a prioritization of aggressive anti-cachexia and nutritional supplementation strategies based on each patient’s underlying disease state and age-related vulnerability.

Pergolotti et al. reported a non-significant but clinically relevant higher proportion of falls in their younger participant cohort (age <65 years: 6.4% vs age ≥65 years: 0%),^12^ and this finding gains greater clarity and context from the current study’s larger dataset. Notably, our larger dataset provides a more robust context for this observation and potentially identifies falls as a significant concern across all adult ages in patients with cancer that is possibly driven by cancer-related toxicities, future treatment-related toxicities, such as chemotherapy-induced peripheral neuropathy, may further increase fall risk,^29–31^ although these were not captured at baseline in our study. Therefore, the current study serves as a crucial validation and substantial expansion of the preliminary findings from smaller and more limited investigations. Its robust statistical power and broad generalizability provide compelling evidence needed to drive significant changes in clinical practice guidelines and resource allocation in oncology.

Importantly, since the GA in this study was conducted prior to antitumor treatment, the observed deficits likely reflect baseline vulnerabilities rather than treatment-related sequelae. These findings likely reflect contributions from both age and disease burden to physiological reserve. Moreover, these results highlight the importance of early pretreatment frailty screening in all adults with cancer, and not just in older adults. Mohile et al. provided strong evidence of GA intervention before cancer treatment for older patients to avoid adverse effects from cancer treatment, reduce falls over 3 months, and less medication use.^32^ Li et al. also conducted a randomized controlled trial in older patients before chemotherapy, which revealed that GA intervention significantly reduced treatment-related adverse effects.^33^ Munsie et al. reported improvements in strength and quality of life after an exercise intervention in young adult patients undergoing treatment.^34^ These studies suggest that GA-guided interventions before cancer treatment are potentially beneficial to patients and even young adults. By validating and extending the findings of previous studies, our analysis provides robust evidence that supports a paradigm shift toward a universal, functionally driven GA in oncology. Such assessments can inform timely interventions and better allocation of supportive care resources across the age spectrum.

Previous studies have consistently demonstrated that baseline frailty is associated with adverse outcomes in cancer, including higher mortality, treatment-related toxicity, and postoperative complications.^3–5^ Moreover, randomized evidence indicates that geriatric-assessment-driven interventions can reduce treatment-related toxicity.^14^^,^^33^ Handforth’s systematic review reported that frailty was significantly independently associated with 5-year all-cause mortality, with a hazard ratio of 1.87.^2^ These findings have important prognostic implications. Previously, frailty was reported to be associated with poor outcomes in patients aged 20-64 years with head and neck cancer, with a higher rate of chemotherapy or incompletion and a higher percentage of hospitalization.^35^ Furthermore, frailty was independently associated with worse overall survival across most age groups, particularly in patients aged 40-79 years. Although the association did not reach statistical significance in the youngest (20-39 years) and oldest (≥80 years) cohorts, potentially because of limited sample sizes or competing mortality risks, the overall trend was clear; higher degrees of frailty at baseline conferred a progressively increased risk of mortality.

In younger patients, interventions may focus on building physiological reserve through prehabilitation, physical conditioning, and nutritional support to mitigate the impact of upcoming intensive treatments such as chemotherapy or chemoradiotherapy.^8^ In older adults, pre-treatment planning may prioritize optimizing comorbidities, reviewing polypharmacy, and addressing baseline cognitive or psychological impairments.^32^ The early and systematic identification of frailty and specific impairments through GA can facilitate timely and appropriate referrals to multidisciplinary teams who provide supportive services, including physical therapy, occupational therapy, nutrition counseling, social work, and psychological support. This necessitates a shift from rigid age-based criteria to a more inclusive functional assessment approach, which has significant implications for resource allocation, healthcare policy, and specialized training of oncology care teams.^36^^,^^37^

Our findings complement and extend prior work by Aleixo and colleagues, who demonstrated that sarcopenia and adverse muscle composition are associated with treatment toxicity and survival outcomes in patients with early-stage breast cancer.^38^^,^^39^ While these studies underscore the importance of physiological vulnerability beyond age, they primarily focused on body composition–based metrics within a single cancer type and specific treatment contexts. Notably, sarcopenia alone did not consistently predict surgical complications, highlighting the limitations of relying on a single-domain vulnerability marker. In contrast, our study applies a comprehensive GA framework across multiple cancer types and a wide adult age range, capturing multidimensional impairments that extend beyond muscle mass alone. The high prevalence of frailty identified even among patients aged 20-39 in our cohort suggests that cancer-related vulnerability is multifactorial and cannot be fully characterized by body composition measures in isolation.

Several limitations merit consideration. First, the use of overall survival in a heterogeneous pan-cancer cohort may obscure cancer-specific prognostic differences. Although analyses were adjusted for cancer type, stage, and treatment modality, residual confounding cannot be excluded, and cancer-specific survival analyses were limited by statistical power. Second, this single-nation Taiwanese study may have limited generalizability to other healthcare systems and cultural contexts, and the cross-sectional design precludes causal inference or assessment of frailty trajectories over time. The relatively high proportion of hematologic malignancies among younger adults may also have influenced frailty prevalence in this group, as suggests some prior reports indicating a high burden of frailty in patients with hematologic malignancies. Finally, detailed treatment-specific, biological, genetic, and socioeconomic determinants of age-specific vulnerability were not captured. Future longitudinal and disease-specific studies are needed to clarify frailty dynamics and evaluate age-tailored interventions.

## Conclusion

This study demonstrates that frailty is a highly prevalent and clinically significant issue across all adult age groups, with the highest rates observed in patients aged ≥80 years. The underlying geriatric assessment (GA)–identified impairments contributing to frailty show distinct age-associated distributions. Younger patients more commonly exhibit nutritional deficits, whereas older patients have a greater burden of functional decline, comorbidities, fall risk, and cognitive impairment. Importantly, both frailty and prefrailty were associated with worse overall survival.

Collectively, these findings provide robust evidence supporting the integration of comprehensive functional assessments, such as GA, into routine oncology care for adult patients regardless of chronological age. This approach facilitates early identification of vulnerability, enables tailored supportive and rehabilitative interventions based on age-related deficit profiles, and has the potential to improve clinical outcomes and quality of life across the cancer continuum. Taken together, our results underscore the need to move beyond a purely age-based framework toward a more functional and individualized paradigm in cancer care.

## Supplementary Material

oyag010_Supplementary_Data

## Data Availability

The datasets used and/or analyzed in the current study are available from the corresponding author upon reasonable request.
