# Supplementary material for: High burden of geriatric assessment impairments across the adult age spectrum in patients with cancer
Source: Oncologist. 2026 Jan 12;31(2):oyag010. doi: 10.1093/oncolo/oyag010 (PMC12861979; doi:10.1093/oncolo/oyag010)
Supplement: oyag010_Supplementary_Data [file oyag010_supplementary_data.docx]

Supplementary Table 1. **Comparison of baseline characteristics between patients aged <65 and ≥65 years**

| Variable, n (%) | All | Age <65, n=1162 | **Age ≥65, n=1339** | P value |
| --- | --- | --- | --- | --- |
| Sex |  |  |  | <0.001 |
| Male | 1664 (66.5) | 867 (74.6) | 797 (59.5) |  |
| Female | 837 (33.5) | 295 (25.4) | 542 (40.5) |  |
| Marriage |  |  |  | 0.08 |
| Married | 2013 (80.5) | 913 (78.6) | 1100 (82.2) |  |
| Educational level |  |  |  | <0.001 |
| Nil or elementary school | 779 (31.1) | 104 (9.0) | 675 (50.4) |  |
| High school | 1228 (49.1) | 756 (65.1) | 472 (35.3) |  |
| College | 451 (18.0) | 274 (23.6) | 177 (13.2) |  |
| Higher than college | 43 (1.7) | 28 (2.4) | 15 (1.1) |  |
| Main caregiver |  |  |  |  |
| Partner/spouse | 1187 (47.5) | 650 (55.9) | 537 (40.1) | <0.001 |
| Current working status |  |  |  | <0.001 |
| Working | 1107 (44.3) | 817 (70.3) | 290 (21.7) |  |
| Current or previous drinking |  |  |  | <0.001 |
| Yes | 832 (33.3) | 507 (43.6) | 325 (24.3) |  |
| Current or previous Smoking |  |  |  |  |
| Yes | 985 (39.4) | 601 (51.7) | 384 (28.7) | <0.001 |
| Cancer type |  |  |  | <0.001 |
| Head and neck | 573 (22.9) | 486 (40.3) | 15 (7.8) |  |
| Esophagus | 217 (8.7) | 167 (14.4) | 50 (3.7) |  |
| Thorax | 118 (4.7) | 61 (5.2) | 57 (4.3) |  |
| Breast | 113 (4.5) | 30 (2.6) | 83 (6.2) |  |
| Stomach or small bowel | 381 (15.2) | 113 (9.7) | 268 (20.0) |  |
| Pancreas | 213 (8.5) | 57 (4.9) | 156 (11.7) |  |
| Liver | 247 (9.9) | 71 (6.1) | 176 (13.1) |  |
| Colorectal | 316 (12.6) | 51 (4.4) | 265 (19.8) |  |
| Hematologic | 205 (8.2) | 97 (8.3) | 108 (8.1) |  |
| Genitourinary | 76 (3.0) | 27 (2.3) | 49 (3.7) |  |
| Others | 42 (1.7) | 20 (1.7) | 22 (1.6) |  |
| ECOG status |  |  |  | <0.001 |
| 0 | 1128 (45.1) | 447 (38.5) | 681 (50.9) |  |
| 1 | 1175 (47.0) | 641 (55.2) | 534 (39.9) |  |
| 2 | 157 (6.3) | 58 (5.0) | 99 (7.4) |  |
| 3 | 36 (1.4) | 12 (1.0) | 24 (1.8) |  |
| 4 | 5 (0.2) | 4 (0.3) | 1 (0.1) |  |
| Tumor stage |  |  |  |  |
| 1 | 203 (8.1) | 43 (3.7) | 160 (11.9) |  |
| 2 | 363 (14.5) | 98 (8.4) | 265 (19.8) |  |
| 3 | 580 (23.2) | 229 (19.7) | 351 (26.2) |  |
| 4 | 1355 (54.2) | 792 (68.2) | 563 (42.0) |  |
| Treatment modality |  |  |  | <0.001 |
| Surgery | 464 (18.6) | 29 (6.3) | 435 (93.8) |  |
| Chemotherapy | 1460 (58.4) | 646 (44.2) | 814 (55.8) |  |
| Concurrent chemoradiotherapy | 577 (23.1) | 487 (84.4) | 90 (15.6) |  |

ECOG, Eastern Cooperative Oncology Group

Categorical variables were compared using the chi-square test or Fisher’s exact test, as appropriate; continuous variables were compared using the independent t-test or Wilcoxon rank-sum test.

Supplementary Table 2. Frailty prevalent rate across different age groups

|  | Full geriatric domain analysis | | |  | Sensitivity analysis (excluding malnutrition) | | |
| --- | --- | --- | --- | --- | --- | --- | --- |
| Age group | Fit | Prefrail | Frail |  | Fit | Prefrail | Frail |
| All patients (n=2501) | 37.1% | 30.9% | 32.0% |  | 17.2% | 32.9% | 49.9% |
| 20-39 (n=100) | 26.0% | 34.0% | 40.0% |  | 42.0% | 38.0% | 20.0% |
| 40-49 (n=260) | 20.0% | 37.7% | 42.3% |  | 44.6% | 35.8% | 19.6% |
| 50-59 (n=510) | 19.6% | 38.2% | 42.2% |  | 45.5% | 29.2% | 25.3% |
| 60-69 (n=795) | 19.4% | 32.5% | 48.2% |  | 38.2% | 31.3% | 30.4% |
| 70-79 (n=660) | 12.9% | 30.9% | 56.2% |  | 31.8% | 29.2% | 38.9% |
| ≥80 (n=176) | 6.8% | 18.8% | 74.4% |  | 14.2% | 28.4% | 57.4% |

Supplementary Table 3. Association between GA-defined frailty and overall survival by age group: full-domain analysis and sensitivity analysis excluding nutritional impairment

|  |  | Full geriatric domain analysis | |  | Sensitivity analysis (excluding malnutrition) | |
| --- | --- | --- | --- | --- | --- | --- |
| Age group | Frailty | Hazard ratio (95% CI) | p value |  | Hazard ratio (95% CI) | p value |
| All patients | Fit | 1 (reference) |  |  | 1 |  |
|  | Prefrail | 1.82 (1.36-2.42) | <0.001 |  | 1.16 (0.96-1.42) | 0.12 |
|  | Frail | 2.73 (2.09-3.57) | <0.001 |  | 1.79 (1.50-2.14) | <0.001 |
| 20-39 (n=100) | Fit | 1 |  |  | 1 |  |
|  | Prefrail | 1.10 (0.38-3.19) | 0.86 |  | 0.66 (0.25-1.71) | 0.39 |
|  | Frail | 1.17 (0.43-3.16) | 0.76 |  | 1.06 (0.41-2.73) | 0.91 |
| 40-49 (n=260) | Fit | 1.00 |  |  | 1 |  |
|  | Prefrail | 2.67 (0.91-7.85) | 0.07 |  | 0.96 (0.0-1.83) | 0.9 |
|  | Frail | 3.50 (1.23-9.93) | 0.02 |  | 1.84 (0.95-3.55) | 0.07 |
| 50-59 (n=510) | Fit | 1.00 |  |  | 1 |  |
|  | Prefrail | 1.49 (0.81-2.75) | 0.20 |  | 0.99 (0.62-1.57) | 0.97 |
|  | Frail | 2.06 (1.15-3.68) | 0.02 |  | 1.53 (0.99-2.36) | 0.06 |
| 60-69 (n=795) | Fit | 1.00 |  |  | 1 |  |
|  | Prefrail | 2.72 (1.13-3.20) | 0.02 |  | 1.27 (0.89-1.82) | 0.19 |
|  | Frail | 3.49 (2.15-5.65) | <0.001 |  | 2.07 (1.49-2.88) | <0.001 |
| 70-79 (n=660) | Fit | 1.00 |  |  | 1 |  |
|  | Prefrail | 1.70 (0.99-2.91) | 0.05 |  | 1.19 (0.84-1.70) | 0.34 |
|  | Frail | 2.31 (1.40-3.81) | 0.001 |  | 1.64 (1.20-2.25) | 0.002 |
| ≥80 (n=176) | Fit | 1.00 |  |  | 1 |  |
|  | Prefrail | 5.66 (0.74-43.1) | 0.09 |  | 1.41 (0.62-3.21) | 0.41 |
|  | Frail | 6.53 (0.91-47.1) | 0.06 |  | 1.76 (0.84-3.73) | 0.14 |

Supplementary Figure 1. Overall survival by frailty status stratified by cancer type


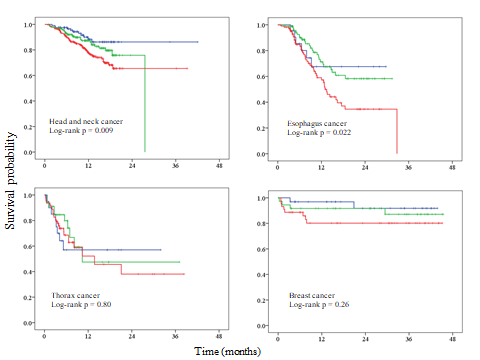


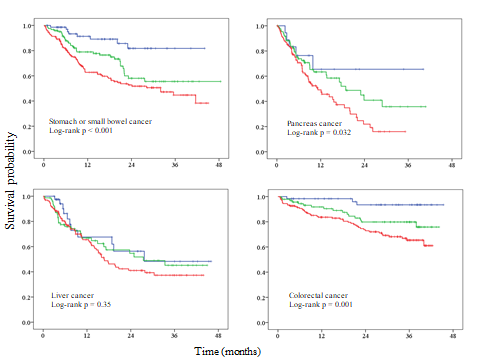

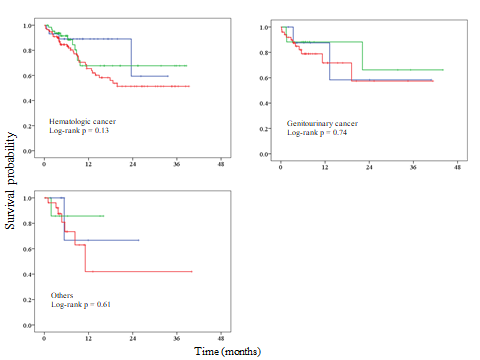


Blue, Green, and Red line indicated fit, prefrial, and fit patient groups, respectively.
